# Post-TTM Rebound Pyrexia after Ischemia-Reperfusion Injury Results in Sterile Inflammation and Apoptosis in Cardiomyocytes

**DOI:** 10.1155/2019/6431957

**Published:** 2019-11-21

**Authors:** Giang Tong, Nalina N. A. von Garlen, Sylvia J. Wowro, Phuong D. Lam, Jana Krech, Felix Berger, Katharina R. L. Schmitt

**Affiliations:** ^1^Department of Congenital Heart Disease/Pediatric Cardiology, Universitäres Herzzentrum Berlin-Medical Heart Center of Charité and German Heart Institute Berlin, Augustenburger Platz 1, 13353 Berlin, Germany; ^2^Department of Pediatric Cardiology, Charité-Universitätsmedizin Berlin, Corporate Member of Freie Universität Berlin, Humboldt-Universität zu Berlin and Berlin Institute of Health, Berlin, Germany

## Abstract

**Introduction:**

Fever is frequently observed after acute ischemic events and is associated with poor outcome and higher mortality. Targeted temperature management (TTM) is recommended for neuroprotection in comatose cardiac arrest survivors, but pyrexia after rewarming is proven to be detrimental in clinical trials. However, the cellular mechanisms and kinetics of post-TTM rebound pyrexia remain to be elucidated. Therefore, we investigated the effects of cooling and post-TTM pyrexia on the inflammatory response and apoptosis in a cardiomyocyte ischemia-reperfusion (IR) injury model.

**Methods:**

HL-1 cardiomyocytes were divided into the following groups to investigate the effect of oxygen-glucose deprivation/reperfusion (OGD/R), hypothermia (33.5°C), and pyrexia (40°C): normoxia controls maintained at 37°C and warmed to 40°C, OGD/R groups maintained at 37°C and cooled to 33.5°C for 24 h with rewarming to 37°C, and OGD/R pyrexia groups further warmed from 37 to 40°C. Caspase-3 and RBM3 were assessed by Western blot and TNF-*α*, IL-6, IL-1*β*, SOCS3, iNOS, and RBM3 transcriptions by RT-qPCR.

**Results:**

OGD-induced oxidative stress (iNOS) in cardiomyocytes was attenuated post-TTM by cooling. Cytokine transcriptions were suppressed by OGD, while reperfusion induced significant TNF-*α* transcription that was exacerbated by cooling. Significant inductions of TNF-*α*, IL-6, IL-1*β*, and SOCS3 were observed in noncooled, but not in cooled and rewarmed, OGD/R-injured cardiomyocytes. Further warming to pyrexia induced a sterile inflammatory response in OGD/R-injured groups that was attenuated by previous cooling, but no inflammation was observed in pyrexic normoxia groups. Moreover, cytoprotective RBM3 expression was induced by cooling but suppressed by pyrexia, correlating with apoptotic caspase-3 activation.

**Conclusion:**

Our findings show that maintaining a period of post-TTM “therapeutic normothermia” is effective in preventing secondary apoptosis-driven myocardial cell death, thus minimizing the infarct area and further release of mediators of the innate sterile inflammatory response after acute IR injury.

## 1. Introduction

Therapeutic hypothermia (TH) is the standard of care for neuroprotection in selected term newborns with hypoxic-ischemic encephalopathy (HIE) and is most effective when applied at 33.5°C for 72 hours [1]. Currently, a targeted temperature management (TTM) of 32-36°C for 24-48 hours is the recommended guideline for mitigating neurological injury in comatose adults with out-of-hospital cardiac arrest [2, 3]. However, the development of fever after rewarming from TTM, termed rebound pyrexia, has been observed in 41% of surviving patients in a multicenter cohort study [4]. They defined pyrexia as a temperature ≥ 38°C within 24 h following rewarming from postarrest TTM, and pyrexia temperature > 38.7°C was associated with worse neurological outcome but not overall lower survival at discharge. Recent randomized TTM control trials even suggest that the prevention of fever or temperature variability by actively maintaining the patient's temperature at 36°C may be just as effective for long-term neurological outcomes as applying mild TH to approximately 33°C [5, 6]. Moreover, Rungatscher et al. observed that postoperative hyperthermia (>37°C) after rewarming from deep hypothermic circulatory arrest was associated with increased morbidity and mortality [7]. While the adverse effects of experimentally induced fever on neuronal damage after global ischemia have been observed [8], the effects of post-TTM rebound pyrexia on ischemia-reperfusion- (IR-) injured cardiomyocytes remain to be elucidated.

Acute myocardial infarction (AMI) has been shown to result in increased expression of proinflammatory cytokines, including tumor necrosis factor- (TNF-) *α*, interleukin- (IL-) 6, and IL-1*β* [9], that can lead to cardiac cell death and dysfunction, as well as ventricular remodeling [10]. Moreover, elevated blood concentrations of IL-6 and TNF-*α* have been reported as independent predictors of mortality in this cohort [11, 12]. Although the majority of proinflammatory cytokines and chemokines are derived from infiltrating monocytes/macrophages to the infarct site after AMI, they are also expressed and secreted by resident cardiac cells [13]. Cardiomyocytes make up 25% of cells in the normal heart and play an active role in mediating innate inflammatory responses, which can result in acute inflammation after IR injury [14]. Therefore, controlling cytokine release from resident cardiomyocytes is a plausible strategy for preventing further tissue damage following prolonged ischemia-reperfusion injury.

We previously demonstrated that IR injury simulated by exposure to oxygen-glucose deprivation (OGD) and subsequent reperfusion (OGD/R) resulted in reduced ATP production, leading to myocardial cell death [15]. Moreover, intra-OGD therapeutic hypothermia (IOTH) attenuated mitochondrial impairment, restored cellular metabolic activity, attenuated cardiomyocyte cell death, and induced RNA binding motif protein 3 (RBM3) expression, a cold shock protein with cytoprotective properties that is expressed in response to hypothermia and various other mild stresses [15, 16]. However, the effect of hypothermia and subsequent rewarming to normothermia or pyrexia on the sterile inflammatory response in an OGD/R cardiomyocyte injury model remains to be elucidated. Therefore, we investigated the efficacy of moderate therapeutic hypothermia (33.5°C) to attenuate the ischemia/reperfusion injury-mediated sterile inflammatory response and the adverse effects of rebound pyrexia in a murine cardiomyocyte model. Additionally, we also investigated the effect of rebound pyrexia on RBM3 expression and further myocardial cell death after an acute ischemia-reperfusion injury.

## 2. Materials and Methods

### 2.1. HL-1 Cell Culture

HL-1 cardiomyocytes are derived from the murine atrial AT-1 tumor cell lineage and were obtained from William C. Claycomb, Ph.D. (LSU Health Sciences Center, New Orleans, LA, USA). They are reported to show spontaneous contractions and a phenotype comparable to adult cardiomyocytes [17] and were cultured following the methods of Krech et al. [16]. Briefly, culture flasks and Petri dishes were precoated with 0.2 *μ*g/cm^2^ fibronectin in 0.02% gelatine for 1 h at 37°C. Cardiomyocytes were cultured at 21% O_2_ and 5% CO_2_ in Claycomb Medium (Sigma-Aldrich), supplemented with 10% FBS (Sigma-Aldrich), 50 *μ*g/ml Primocin (InvivoGen), 2 mM L-glutamine (Merck Millipore), and 0.1 mM norepinephrine (Sigma-Aldrich). Cells were passaged upon reaching 90% confluency at 1 : 2 to 1 : 5 using trypsin/EDTA (0.05/0.02%, respectively; Biochrom). HL-1 cardiomyocytes were divided into the following groups to investigate the effect of OGD/R, hypothermia (33.5°C), and pyrexia (40°C): normoxia control groups maintained at 37°C and warmed to 40°C, OGD/R groups maintained at 37°C and cooled to 33.5°C for 24 hours with subsequent rewarming to 37°C, and OGD/R pyrexia groups further warmed from 37 to 40°C.

### 2.2. Oxygen-Glucose Deprivation/Reperfusion (OGD/R)

Ischemia-reperfusion injury was simulated *in vitro* by exposure to OGD/R, as previously established in our laboratory [16]. Briefly, HL-1 cardiomyocytes were deprived of oxygen and glucose for 6 hours in glucose/serum-free DMEM (Biochrom) at 0.2% O_2_ and 5% CO_2_ in a CO_2_ incubator (Binder) [15]. Control groups were kept at normoxia (21% O_2_) in DMEM containing glucose (Biochrom) and 10% FBS (Biochrom). After 6 h of OGD, reperfusion was simulated by restoration of nutrients in complete Claycomb Medium (Sigma-Aldrich) and 21% O_2_ in all the groups. All experimental media were supplemented with 50 *μ*g/ml Primocin (InvivoGen) and 2 mM L-glutamine (Merck Millipore).

### 2.3. Targeted Temperature Management (TTM)

**Figure 1:**
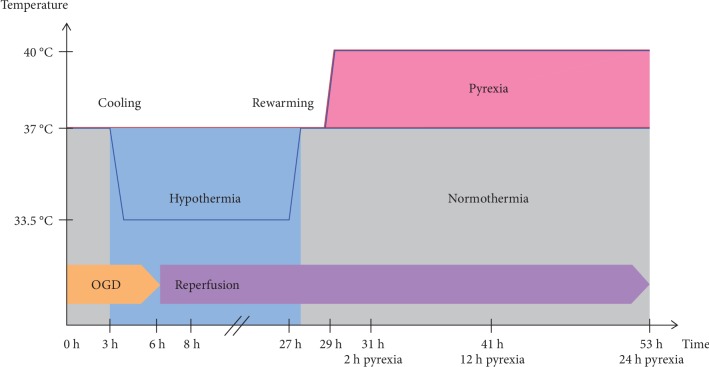
Experimental time-temperature protocol. Normothermic OGD/R-injured groups were maintained at 37°C for the duration of the experiment, while TTM groups were cooled to 33.5°C after 3-hour exposure to OGD and maintained during simulated reperfusion for 24 hours. All experimental cooled groups were then rewarmed to and maintained at 37°C. Cooled pyrexia groups were maintained at 37°C for only 2 hours and then along with normothermic pyrexia groups further warmed to 40°C at 29 h after experimental start and maintained for an additional 24 hours. Samples were analyzed directly after OGD (6 h), 2 hours into the early reperfusion phase (8 h), the end of the cooling phase (27 h), 2 hours after rewarming to normothermia (29 h), and then 2, 12, and 24 hours after initiation of pyrexia (31, 41, and 53 h after experimental start, respectively).

We previously established a time-temperature protocol for intraischemic cooling (33.5°C) for the HL-1 cardiomyocytes, based on the guidelines from the European Resuscitation Council for cardiac arrest survivors (see Figure 1) [15, 18]. Briefly, normothermic OGD/R-injured groups were maintained at 37°C for the duration of the experiment, while TTM groups were cooled to 33.5°C after 3-hour exposure to OGD and maintained during simulated reperfusion for 24 hours. All experimental cooled groups were then rewarmed to and maintained at 37°C. Cooled pyrexia groups were maintained at 37°C for only 2 hours, then along with normothermic pyrexia groups further warmed to 40°C at 29 h after experimental start and maintained for an additional 24 hours. Samples were analyzed directly after OGD (6 h), 2 hours into the early reperfusion phase (8 h), the end of the cooling phase (27 h), 2 hours after rewarming to normothermia (29 h), and 2, 12, and 24 hours after initiation of pyrexia (31, 41, and 53 h after experimental start, respectively) in order to thoroughly investigate the effects of OGD/R, TTM, and pyrexia on the cardiomyocytes.

### 2.4. Protein Extraction and Western Blot Analysis

Caspase-3 activation and RBM3 expression were assessed by Western blot following the methods of Krech et al. [16]. Briefly, HL-1 cardiomyocytes were seeded onto 22 cm^2^ cell culture dishes at a density of 5 × 10^5^ cells per dish 48 h before conducting the experiments as described above. Attached cells were mechanically scratched off the plate surface and lysed using a modified RIPA buffer (50 mM Tris-HCl, pH 7.5), 150 mM sodium chloride, 1% Triton X-100, 0.1% sodium dodecyl sulfate, 0.5% Na-deoxycholate, 2 mM ethylenediaminetetraacetic acid, 1 mM phenylmethylsulfonyl fluoride, sodium fluoride, and protease inhibitor cocktail 3 (all from Sigma-Aldrich) and quantified using a BCA-Protein Assay Kit (Pierce Biotechnology). Protein extracts (30 *μ*g) were electrophoresed on 15% SDS polyacrylamide gels and transferred to PVDF membranes. Membranes were blocked with 5% nonfat dried milk powder in Tris-buffered saline+0.1% Tween-20 and incubated with anti-caspase-3 (1 : 500) and anti-RBM3 (1 : 1000) or blocked with 5% BSA for incubation with anti-*β*-actin (1 : 15,000) at 4°C overnight. All primary antibodies were rabbit polyclonals purchased from Cell Signaling Technology. An HRP-conjugated donkey anti-rabbit secondary antibody (Dianova) was incubated for 1 h and detected with SuperSignal™ West Dura Chemiluminescent Substrate (Pierce Biotechnology). Densitometry quantification of the Western blots was performed using Image Lab (Bio-Rad Laboratories) and normalized to *β*-actin for equal protein loading.

### 2.5. RNA Isolation and RT-qPCR

Sterile inflammatory response was assessed by real-time quantitative PCR (RT-qPCR). Total RNA from HL-1 cardiomyocytes was isolated using the GeneMatrix Universal RNA Purification Kit (Roboklon) according to the manufacturer's instructions. RNA concentration and purity were determined by spectrophotometric measurements at 260 and 280 nm using NanoDrop 2000 (NanoDrop) and agarose gel electrophoresis. cDNA was transcribed from 1.5 *μ*g total RNA using a High-Capacity cDNA Reverse Transcription Kit (Applied Biosystems) using a PTC200 Thermal Cycler (MJ Research). Expression of target genes and the endogenous control glyceraldehyde 3-phosphate dehydrogenase (GAPDH) was assessed by real-time qPCR using the TaqMan® Gene Expression Assays (see Table 1) and StepOnePlus™ Real-Time PCR System (Applied Biosystems) according to the manufacturer's recommendations. Reactions with no reverse transcripts and templates were included as negative controls. Relative quantification of gene expression was normalized to the housekeeping gene GAPDH, using the 2^-*ΔΔ*ct^ method, and illustrated as fold change [15].

### 2.6. Statistical Analysis

Data were analyzed and graphed using GraphPad Prism 5 (GraphPad Software). Results were expressed as means ± standard deviations. Experiments were independently repeated at least three times. One-way ANOVA followed by Tukey's posttest was used for multiple group comparison, and *p* < 0.05 was considered statistically significant.

## 3. Results

### 3.1. OGD/R Induces Oxidative Stress in HL-1 Cardiomyocytes

**Figure 2:**
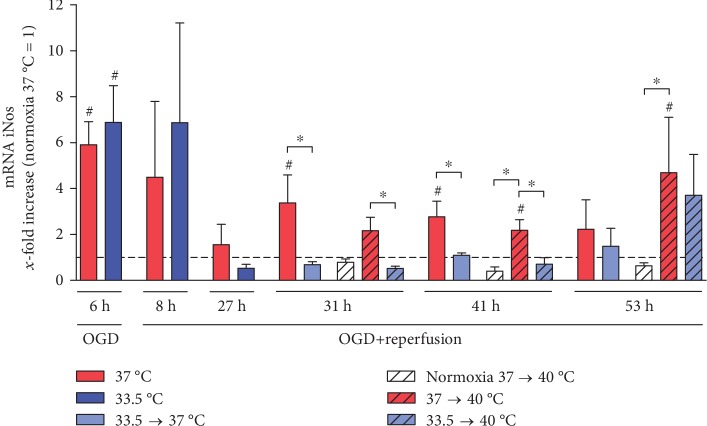
Hypothermia attenuated OGD/R- and pyrexia-induced iNOS expression in the HL-1 cardiomyocytes in the late reperfusion and pyrexia phase (31–53 h). Data from 3 to 5 independent experiments is presented as mean ± SD. ^∗^*p* ≤ 0.05 and ^#^*p* ≤ 0.05 as compared to normoxia control at 37°C (normalized to 1).

We investigated the effect of exposure to OGD/R, hypothermia, and pyrexia on the inducible NO synthase (iNOS) expression in the HL-1 cardiomyocytes (see Figure 2) and observed a significant increase in iNOS expression relative to normoxia control after exposure to OGD that was not attenuated by the brief period of hypothermia (6 h), but no significant increases were observed in the reperfusion phase (8–27 h). Even after posthypothermia rewarming to 37°C, iNOS transcription stayed significantly attenuated by cooling compared to noncooled OGD/R groups (29–41 h). Further warming to pyrexia also resulted in a significant increase in iNOS expression (31-53 h) that was attenuated by cooling in the early pyrexia phase (31-41 h), but not after 24 hours (53 h). Interestingly, exposure to pyrexia alone did not induce increased iNOS transcription in the undamaged control cardiomyocytes that were warmed to pyrexia.

### 3.2. OGD/R-Induced Sterile Inflammatory Response Is Exacerbated by Pyrexia

**Figure 3:**
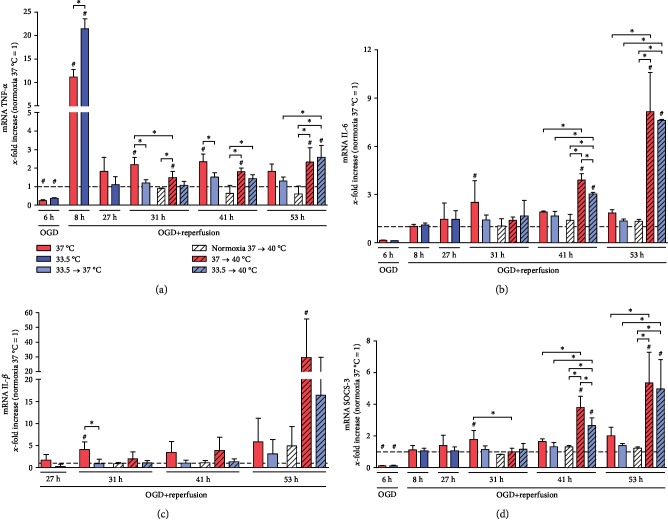
(a) TNF-*α* expression was suppressed by OGD (6 h). OGD/R-induced damage leads to a significant increase in TNF-*α* expression relative to normoxia control in the early reperfusion phase (8 h), which was significantly higher in the cooled than in the noncooled group. During late reperfusion (31 and 41 h), however, the noncooled OGD/R-injured group stayed significantly elevated, whereas the cooled group showed no such effect. Further warming to pyrexia induced TNF-*α* expression in OGD/R-injured groups irrespective of previous temperature management. (b) IL-6 expression was suppressed by OGD (6 h), and hypothermia temporarily attenuated pyrexia-induced IL-6 expression in OGD/R-injured cardiomyocytes (41 h). (c) Pyrexia increased IL-1*β* expression in noncooled OGD/R-injured cardiomyocytes that was not attenuated by hypothermia (53 h). (d) SOCS-3 expression was significantly inhibited by OGD (6 h) and increased during late reperfusion (31 h) in the noncooled OGD/R-injured groups. Warming to pyrexia significantly induced SOCS-3 expression in both cooled and noncooled OGD/R-injured cardiomyocytes and was briefly attenuated by hypothermia (41 h). Data from 3 to 5 independent experiments is presented as mean ± SD. ^∗^*p* ≤ 0.05 and ^#^*p* ≤ 0.05 as compared to normoxia control at 37°C (normalized to 1).

We investigated the effect of hypothermia and subsequent warming to pyrexia on OGD/R-induced TNF-*α* (see Figure 3(a)), IL-6 (see Figure 3(b)), and IL-1*β* (see Figure 3(c)) expression, as well as the negative regulator of cytokine signaling, SOCS-3 (see Figure 3(d)), in the HL-1 cardiomyocytes. A significant decrease in TNF-*α* transcription relative to normoxia control was observed after exposure to OGD (6 h) that was followed by a significant spike in the early reperfusion phase, which was augmented by cooling (8 h). TNF-*α* transcription eventually diminished to normoxia control levels in the cooled groups (27–53 h), but stayed significantly higher in the noncooled groups at the later reperfusion time points (31-41 h). Warming OGD/R-injured cardiomyocytes to pyrexia also resulted in significantly higher TNF-*α* transcription relative to normoxia controls at 37°C as well as normoxia groups warmed to pyrexia (31–53 h), but not to the OGD/R-injured groups that were either maintained at or rewarmed to 37°C (31-41 h). Additionally, no significant attenuations by cooling were observed in the OGD/R-injured groups after 24-hour exposure to pyrexia (53 h).

Similar to TNF-*α*, IL-6 transcription was also significantly suppressed relative to normoxia control by exposure to OGD (6 h). Unlike TNF-*α*, IL-6 transcription did not peak in the reperfusion phase (8-29 h). A brief increase in IL-6 transcription was observed in the noncooled OGD/R group, but not in the cooled OGD/R group in the late reperfusion phase (31 h). Further warming to pyrexia resulted in the greatest increases in IL-6 transcriptions in both cooled and noncooled OGD/R groups relative to both normoxia control and OGD/R groups maintained at or rewarmed to 37°C (41 and 53 h). Even though previous cooling attenuated this increase in IL-6 after 12-hour exposure to pyrexia (41 h) in the cooled OGD/R group compared to the noncooled OGD/R group, this protective effect was no longer observed after 24-hour exposure to pyrexia (53 h). Pyrexia alone however did not induce IL-6 expression in the undamaged normoxia control cardiomyocytes.

The expression of IL-1*β* was observed to be comparable to IL-6 expressions in all experimental groups during the OGD/R phase and was not significantly induced by hypothermia. However, a significantly lower IL-1*β* transcription was observed in the cooled OGD/R group rewarmed to 37°C relative to the noncooled OGD/R-injured group (31 h). Moreover, warming to pyrexia resulted in a significant increase in IL-1*β* transcription in the noncooled OGD/R-injured group (53 h).

Suppressor of cytokine signaling 3 (SOCS-3) gene expression was significantly decreased by OGD (6 h) relative to normoxia control, recovered to normoxia level in the reperfusion phase, and was significantly induced in the noncooled OGD/R-injured group but not in the cooled groups (29 and 31 h). Rewarming to pyrexia, however, induced significant increases in SOCS-3 transcription in the OGD/R-injured cardiomyocytes compared to both normoxia control and corresponding OGD/R-injured groups maintained at or rewarmed to 37°C (41 and 53 h), which was briefly attenuated by previous cooling after 12-hour exposure to pyrexia (41 h). Interestingly, no significant increased SOCS-3 expression was observed in the undamaged normoxia control cardiomyocytes warmed to 40°C.

### 3.3. Cold Shock RBM3 Is Induced by Hypothermia and Suppressed by Pyrexia

**Figure 4:**
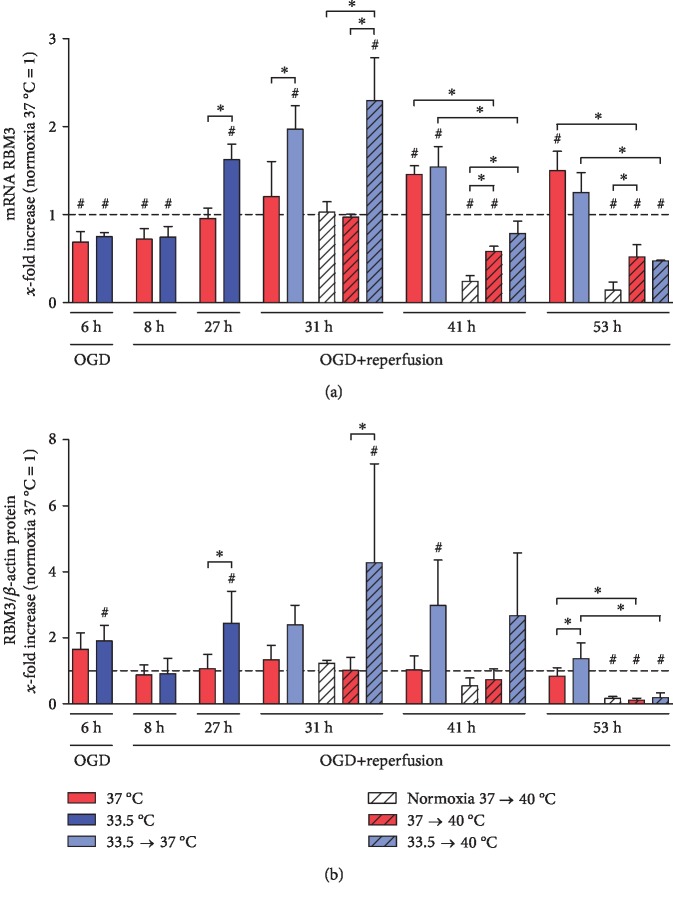
Hypothermia induces while pyrexia inhibits RBM3 (a) mRNA transcriptions and (b) intracellular protein levels in HL-1 cardiomyocytes. Data from 3 to 5 independent experiments is presented as mean ± SD. ^∗^*p* ≤ 0.05 and ^#^*p* ≤ 0.05 as compared to normoxia control at 37°C (normalized to 1).

Exposure to moderate hypothermia for 24 hours significantly induced both RBM3 mRNA and protein expressions in the HL-1 cardiomyocytes (27 h) (see Figure 4). Induced RBM3 expression was observable up to 14 hours after rewarming to normothermia (37°C at 29, 31, and 41 h), 2 hours after further warming to pyrexia (31 h), and gradually returned to baseline levels after 24 hours. However, prolonged exposure to pyrexia for 24 hours resulted in a significant suppression of RBM3 expression in all groups at the mRNA and protein levels (53 h).

### 3.4. Pyrexia Induces Apoptosis in OGD/R-Injured Cardiomyocytes

**Figure 5:**
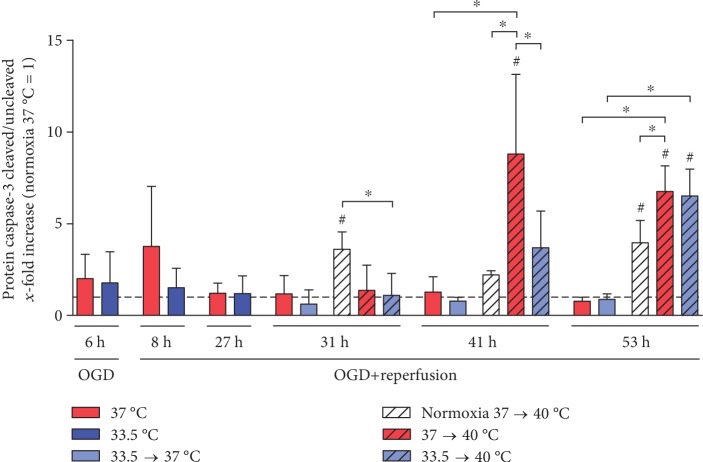
Pyrexia induces caspase-3 cleavage in the OGD/R-injured cardiomyocytes (41 and 53 h) that was briefly attenuated by hypothermia (41 h). Undamaged normoxia control cardiomyocytes warmed to pyrexia also showed increased cleavage of caspase-3 (31 and 53 h). Data from 3 to 4 independent experiments is presented as mean ± SD. ^∗^*p* ≤ 0.05 and ^#^*p* ≤ 0.05 as compared to normoxia control at 37°C (normalized to 1).

Further warming to fever induced a secondary cell death mechanism in the cardiomyocytes exposed to OGD/R. We observed significant increases in caspase-3 activation, a hallmark of the apoptosis programmed cell death mechanism, in OGD/R-injured cardiomyocytes after warming to pyrexia at 41 h and 53 h (see Figure 5). Previous treatment with cooling could temporarily attenuate caspase-3 cleavage at 41 h but could not maintain protection for a prolonged exposure to pyrexia (53 h). Pyrexia in noninjured cardiomyocytes also led to apoptosis (31 and 53 h), but to a significantly lesser extent than in the OGD/R-injured cells (41 and 53 h). Rewarming of the OGD/R-injured cardiomyocytes to normothermia however did not result in increased activation of caspase-3.

## 4. Discussion

Ischemia-reperfusion injury causes myocardial cell death by inducing intracellular calcium overload, oxidative stress, and inflammation, which can be exacerbated by pyrexia. IR induces necrotic cell death during the ischemic phase followed by ATP-dependent apoptotic signaling cascades during the reperfusion phase, leading to an apoptosis-induced secondary cell death that can account for up to 50% of the infarct area [16]. Correspondingly, we previously observed that exposure to OGD induces mitochondrial dysfunction and cell death in the HL-1 cardiomyocytes that could be attenuated by hypothermia [15, 16]. OGD/R as well as changes in temperature can cause increased production of reactive oxygen species or free radicals, resulting in oxidative stress and terminal apoptosis and/or cell death [19]. In correlation with previous findings, we observed an increase in OGD/R-induced iNOS transcription that was also attenuated by cooling in the HL-1 cardiomyocytes, presumably due to the inhibition of nuclear factor kappa B (NF-*κ*B) translocation to the nucleus [20].

While necrosis is generally observed after an acute ischemic incident, apoptosis is the primary myocardial cell death mechanism following reperfusion. We did not observe the induction of apoptosis in the reperfusion phase, but warming to pyrexia after OGD/R with or without hypothermia resulted in the induction of apoptosis, as evidenced by significant increases in the cleavage of caspase-3 (53 h). Unlike necrosis, apoptosis can have beneficial effects and be reversed by the activation of prosurvival pathways, including the Janus kinase- (JAK-) STAT signaling pathway in which cardiac-specific SOCS-3 plays a key role in promoting myocardial IR-induced injury [21]. Nagata et al. observed that induced cardiac-specific SOCS-3 expression correlated with decreased activation of prosurvival STAT3, AKT, and ERK1/2, as well as decreased expression of myeloid cell leukemia-1 (Mcl-1), a member of the antiapoptotic Bcl-2 family. Moreover, they also observed significantly reduced cleavage of caspase-3 and smaller infarct sizes in cardiac-specific SOCS-3-KO mice at 6 hours and 24 hours after reperfusion, respectively [22]. This is in correlation with our findings that pyrexia induces SOCS-3 expression, resulting in increased cleavage of caspase-3, which could be temporarily attenuated by hypothermia. We previously observed that hypothermia significantly increased the Bcl-2/Bax ratio to protect OGD/R-injured HL-1 cardiomyocytes from apoptosis [16] but did not observe any significant increases upon warming to pyrexia (data not shown). However, the expression of Mcl-1 warrants further investigation as a key STAT3 activator gene of apoptosis after myocardial IR-induced injury.

Moreover, our observation of suppressed RBM3 expression by pyrexia in the HL-1 cardiomyocytes corresponds with previous findings that showed that blood RBM3 mRNA levels were also decreased in febrile children [23]. RBM3 has been shown *in vitro* to have antiapoptotic effects in a variety of cellular stress situations, including OGD/R, staurosporine, H_2_O_2_, and nitric oxide (NO) treatment, by attenuating caspase-3 activation and PARP cleavage, as well as inducing Bcl-2 expression [24–26]. Our observation of increased caspase-3 activation in conjunction with suppressed RBM3 expression by pyrexia in OGD/R-injured cardiomyocytes further supports the cytoprotective properties of RBM3 and warrants further investigation as a promising therapeutic strategy against IR injury.

**Figure 6:**
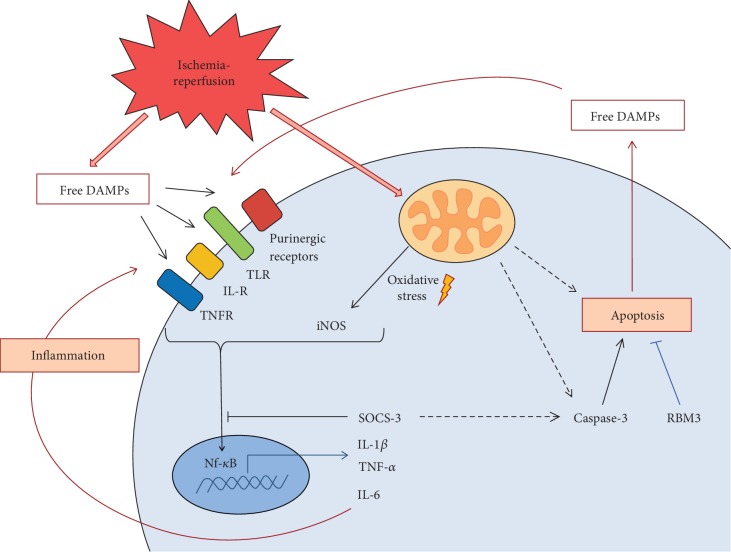
Synopsis of the sterile inflammatory response and myocardial apoptotic cell death induced by ischemia-reperfusion injury, hypothermia, and post-TTM rebound pyrexia in the HL-1 cardiomyocytes.

The heart is normally not a key source of inflammatory cytokines and therefore is not considered an immunologically active organ [27]. However, a variety of stresses, including infection by pathogens, mechanical stretch, oxidative stress, and ischemia, can induce innate immune responses that can lead to acute inflammation, and the extent of the inflammatory response after an acute ischemic incident is a key factor that dictates the severity of damage to cardiac tissue. Moreover, IR injury induces the release of host damage-associated molecular patterns (DAMPs) into the extracellular matrix where they bind to various pattern recognition receptors (PRRs) on the surface of neighboring structural cardiac cells, such as cardiomyocytes, endothelial cells, and fibroblasts, or recruited immune cells to also activate endogenous inflammatory signaling cascades (see Figure 6). This activates various signaling transcription factors, in particular NF-*κ*B, to induce the expression of proinflammatory cytokines, including IL-1*β*, IL-18, IL-6, and TNF-*α* [28].

In correlation with previous reported findings [27], we did not observe significant changes in IL-1*β* transcription after exposure to OGD/R and hypothermia followed by rewarming to normothermia. However, we did observe significant increases in IL-1*β* transcription after prolonged exposure to pyrexia (53 h), which could be attenuated by preceding hypothermia. Interestingly, this pyrexia-induced expression of IL-1*β* correlates with the significant induction of IL-6 transcription observed at the same time point after warming to pyrexia. Our findings further support previous reports of increased IL-6 expression in cardiomyocytes in response to increased IL-1*β* [28], which acts to recruit leukocytes and propagates inflammation in the heart [29]. We also observed a tendency towards increased MCP-1/CCL2 transcription after warming to pyrexia, though not to significance, that also plays a role in regulating leukocyte trafficking (data not shown).

IL-6 has been shown to have cardioprotective effects [29], but chronic or excessive expression of IL-6 can be fatal and has been shown to cause heart failure in a rodent model [30]. Additionally, IL-6 along with IL-1*β* and TNF-*α* has been known to act as endogenous pyrogens, thus contributing to the induction of fever [31]. We observed that cooling effectively maintained IL-6 transcription at normoxia control levels at all investigated time points and throughout rewarming to 37°C. Therefore, attenuating IL-6 expression in cardiomyocytes may be an essential strategy to minimize the systemic inflammatory response often referred to as rebound pyrexia in hypothermia-treated cardiac arrest patients.

SOCS-3 is a member of the STAT-induced STAT inhibitor (SSI) family that functions as a negative regulator of cytokine signaling to control immune homeostasis in both physiological and pathological conditions. It therefore plays an important role in restraining inflammation, yet allowing optimal immune response against infections. However, similar to the findings of Nagata et al., we also observed significant increases in TNF-*α*, IL-6, and IL-1*β* transcriptions relative to normoxia control that correlated with significant increases in SOCS-3 in the OGD/R groups upon warming to pyrexia [20], whereas previously cooled OGD/R groups rewarmed to normothermia did not show this inflammatory response and even resulted in attenuated IL-1*β* expression.

Limitations of our study lie in the use of a cardiomyocyte monoculture model, as our focus was to investigate the specific contribution of resident cardiomyocytes to the inflammatory response. Of course the interaction between leukocytes, cardiac fibroblasts, and resident cardiomyocytes plays an important role in the inflammatory response after IR-induced cardiac injury and warrants further investigation. Moreover, the release of cardiac-specific DAMPs from necrotic myocardial cells was not addressed in this study but is currently under investigation in a primary murine cardiomyocyte model in our lab.

## 5. Conclusion

Targeted temperature management is an effective therapeutic strategy for ischemia/reperfusion injury, but preventing post-TTM rebound pyrexia is crucial to minimizing the sterile inflammatory response and subsequent cardiomyocyte apoptosis after an acute ischemia-reperfusion injury. Optimization of the TTM protocol for postcardiac arrest care is currently a topic of great research interest. Although most efforts are focused on the application of TTM, including optimal cooling temperature, rates of cooling and subsequent rewarming, practical methods of cooling that allow for adequate and consistent temperature control, and eligible patient cohort, preventing the onset of post-TTM rebound pyrexia warrants further investigation. Our findings show that maintaining a period of post-TTM normothermia, referred to as “therapeutic normothermia” by Leary et al., is effective in preventing secondary apoptosis-driven myocardial cell death, thus minimizing the infarct area and further release of various mediators of the innate sterile inflammatory response after an acute ischemia/reperfusion injury.

## Figures and Tables

**Table 1:** TaqMan® Gene Expression Assays.

Gene	Assay ID
GAPDH	99999915_g1
IL-1*β*	00434228_m1
IL-6	00446190_m1
iNOS	00440502_m1
RBM3	01609819_g1
SOCS3	00545913_s1
TNF-*α*	00443260_g1

## Data Availability

The (experimental) data used to support the findings of this study are available from the corresponding author upon request.

## Abbreviations

AMI: Acute myocardial infarction
AKT: Protein kinase B
ATP: Adenosine triphosphate
Bax: Bcl-2-associated X protein
Bcl-2: B-cell lymphoma 2
BSA: Bovine serum albumin
DAMPs: Damage-associated molecular patterns
EDTA: Ethylenediaminetetraacetic acid
ERK1/2: Extracellular signal-regulated protein kinases 1 and 2
FBA: Fetal bovine serum
GAPDH: Glyceraldehyde-3-phosphate dehydrogenase
HIE: Hypoxic/ischemic encephalopathy
IL-1*β*/-6/-18: Interleukin-1*β*/-6/-18
iNOS: Inducible nitric oxide synthase
IOTH: Intra-OGD therapeutic hypothermia
IR: Ischemia-reperfusion
JAK-STAT: Janus kinase/signal transducers and activators of transcription
Mcl-1: Myeloid cell leukemia-1
MCP-1/CCL2: Monocyte chemoattractant protein-1/CC-chemokine ligand 2
(m)RNA: (Messenger) ribonucleic acid
NF-*κ*B: Nuclear factor kappa B
NO: Nitric oxide
OGD/R: Oxygen-glucose deprivation/reperfusion
PARP: Poly(ADP-ribose) polymerase 1
PRRs: Pattern recognition receptors
PVDF: Polyvinylidene difluoride
RBM3: RNA binding motif 3
RIPA buffer: Radioimmunoprecipitation assay buffer
SDS: Sodium dodecyl sulfate
SOCS-3: Suppressor of cytokine signaling 3
STAT3: Signal transducer and activator of transcription 3
TH: Therapeutic hypothermia
TNF-*α*: Tumor necrosis factor-alpha
TTM: Targeted temperature management.
